# Cardiovascular risk factors in diabetic patients with and without metabolic syndrome: a study based on the Rafsanjan cohort study

**DOI:** 10.1038/s41598-022-27208-5

**Published:** 2023-01-11

**Authors:** Gholamreza Bazmandegan, Mitra Abbasifard, Ali Esmaeili Nadimi, Hasan Alinejad, Zahra Kamiab

**Affiliations:** 1 Clinical Research Development Unit, Ali-Ibn Abi-Talib Hospital, Rafsanjan University of Medical Sciences, Rafsanjan, Iran; 2 Department of Family Medicine, Ali-Ibn Abi-Talib Hospital, School of Medicine, Rafsanjan University of Medical Sciences, Rafsanjan, Iran; 3 Non-Communicable Diseases Research Center, Rafsanjan University of Medical Sciences, Rafsanjan, Iran; 4 Department of Internal Medicine, Ali-Ibn Abi-Talib Hospital, School of Medicine, Rafsanjan University of Medical Sciences, Rafsanjan, Iran

**Keywords:** Cardiology, Diseases, Endocrinology, Health care, Medical research, Risk factors

## Abstract

Cardiovascular disease (CVD) is the leading cause of death and disability in people with diabetes mellitus (DM), since finding the correlation between DM and CVD risk factors can be effective in preventing the incidence of morbidity and mortality in patients. This study aimed to determine the prevalence of cardiovascular risk factors in people with and without metabolic syndrome (MtS) in DM. This cross-sectional study was part of the Rafsanjan Cohort Study as part of the comprehensive Persian (Prospective Epidemiological Research Studies in IRAN) on 35–70-year old adults with and without MtS in DM. Indicators of CVD risk factors, including gender, age, blood pressure, dyslipidemia, smoking, alcohol consumption, fasting blood sugar, creatinine, blood urea, waist circumference, body mass index, family history, physical inactivity, and fruit and vegetable consumption, were collected in the Persian Cohort Questionnaire. The data was analyzed by SPSS software version 22. The prevalence of MtS in 1933 participants was estimated to be 80% (95% confidence interval 78.1–81.8%). In the logistic regression model, smoking, alcohol consumption, and triglycerides were identified as the factors associated with MtS. Our results show that, based on our study, the prevalence of cardiovascular risk factors in DM was high. The suggested solutions in this field are to reduce smoking and alcohol consumption, as well as to control hypertension, hyperlipidemia, and being overweight.

## Introduction

According to the International Diabetes Federation (IDF), diabetes mellitus (DM) affects 8.8% of the world's population and is expected to rise to 642 million by 2040^1^ The prevalence of DM is constantly increasing over time^1,2^ In Iran, the prevalence of DM in adults aged 25 to 70 years is reported to be 11.9%. It is estimated that by 2030, about 9.2 million Iranians are likely to have DM^2^ Cardiovascular disease (CVD) is one of the leading causes of death and disability in people with DM^1,2^ DM directly and indirectly increases the risk of CAD. The increased risk factor for CAD associated with DM is not well described^1,2^ The risk of CVD is constantly increasing by increasing fasting plasma glucose levels, even before they reach a sufficient level to diagnose DM^3^ DM reduces life expectancy by up to 10 10 years, and more than 50% of patients die of a cardiovascular event^4^ People with DM are more likely to be affected by CVD than non-diabetic people^2,5^

Diabetic patients had a 10% higher risk of CVD, a 53% higher risk of MI, a 58% higher risk of stroke, and a 12% higher risk of heart failure than the non-diabetic population. Thus, DM is a major risk factor for CVD and its consequences^6^ The literature review shows that in New York, patients with DM are almost three times more likely to develop heart disease than their non-diabetic counterparts^7^ This ratio has been studied in some areas in India. For example, in a study in Yazd, the risk of CVD in DM patients was about 2–4 times^8^ that of the general population, while in another study in Ahvaz it was 2–8 times that of the general population^9^.

The risk of CVD in DM follows a slope, and the severity of this slope depends on a combination of multiple risk factors^10^ Most of these additional risks of CVD in DM are associated with an increased prevalence of known risk factors, such as hypertension, dyslipidemia, and obesity^11^ Over the last decade, studies have shown that treating known risk factors for patients with DM is extremely important in reducing the risks of CVD^12^ Poor control of most cardiovascular risk factors has been observed in the diabetic population. However, the additional risks of CVD in DM patients cannot be attributed solely to the higher prevalence of known risk factors^13^ Therefore, other risk factors may be important in people with DM^14^ A set of interrelated risk factors characterizes the metabolic syndrome (MtS), including hypertension, hyperglycemia, abdominal obesity, and dyslipidemia^15^ The disease is associated with an increased risk of cardiovascular events, DM, and deaths^16^.

NCEP-ATPIII (National Cholesterol Education Program Adult Treatment Panel III) Criteria, IDF, and WHO (World Health Organization) definitions reported that the prevalence of MtS in DM was 45.8%, 57.7%, and 28%, respectively, in India^17^ According to a study conducted in Nepal, the total age-adjusted prevalence rates of MtS according to Harmonized, NCEP ATP III, WHO, and IDF definitions were 80.3%, 73.9%, 69.9%, and 66.8%, respectively. The lowest overall agreement was observed between WHO and IDF definitions, and the highest overall agreement was between Harmonized and NCEP ATP III definitions^18^.

The extent of these risk factors has been widely examined in studies, since finding the correlation between DM risk factors and CVD can be effective in preventing the incidence of morbidity and mortality in patients^19^ Endocrinologists and cardiologists suggest that more efforts should be made to improve risk factors for heart disease in diabetic patients due to their higher risk of heart attack and the higher mortality^20^ As a result, the purpose of this study was to determine the prevalence of cardiovascular risk factors in people with and without MtS in DM in the Rafsanjan adult cohort study.

## Methods

### Data sources

This cross-sectional study was performed based on Rafsanjan Cohort Study (RCS)^21^ as part of the comprehensive Persian (Prospective Epidemiological Research Studies in Iran)^22^ The cohort study included 9,990 subjects aged 35–70 years who were enrolled in the Rafsanjan Cohort Study (RCS), (21), which included both urban and suburban Rafsanjan areas in southern Iran. The following were the cohort study's inclusion criteria: 1- Iranian citizenship 2- having an age range of 35–70 years, and 3- living at least 9 months a year in the studied area in Rafsanjan city. The exclusion criteria included a lack of understanding of the Iranian language and the existence of severe physical and mental disorders. Persian Cohort standard questionnaires consisting of 482 questions in 3 major sections of general, medical, and nutrition were asked of the participants by a trained interviewer. The validity and reliability of all questionnaires were confirmed. The face-to-face interview was conducted by trained interviewers and the participants’ answers were collected electronically and confidentially after obtaining their consent^21^.

### Variables of interest and measurements

In this study, all the DM patients in the cohort population were included based on their past medical history, self-expression and confirmatory tests. The DM cases were considered valid if they were diagnosed by a healthcare provider. Each individual's MtS status was determined, and they were divided into two groups: those who had MtS and those who did not. The diagnostic criteria for this syndrome were designed so that the patient met at least three of the five MtS criteria described by the American Heart Association^23^ at the same time, including: (a) central obesity defined as a waist circumference of 88 cm (35 inches) or greater in women and 102 cm (40 inches) in men; (b) fasting serum triglyceride level equal to or greater than 150 mg/dL or on hypertriglyceridemia drug therapy (e.g., fibrates, nicotinic acid); (c) High Density Lipoprotein (HDL) level less than 50 mg/dL in women and less than 40 mg/dL in men, or on drug therapy for low HDL level (fibrates, nicotinic acid); (d) elevated diastolic blood pressure equal to or greater than 85, or elevated systolic blood pressure equal to or greater than 130, or on hypertension medication; (e) a fasting glucose level of 100 mg/dL or higher, or being treated with medication for hyperglycemia or diabetes.

Individuals' demographic and clinical characteristics, such as gender, age, education level, residence, race, hypertension, dyslipidemia, smoking, alcohol consumption, systolic and diastolic blood pressure, heart rate, fasting blood sugar, triglycerides, cholesterol (LDL, HDL), creatinine, blood urea nitrogen (BUN), alkaline phosphatase (ALP), waist circumference, body mass index (BMI = weight (kg)/height2 (m)), height and weight, family history of cardiovascular disease, physical inactivity, and insufficient consumption of fruits and vegetables were extracted from the cohort center database.

The ratio of triglyceride to high-density lipoprotein cholesterol (TG/HDL-C) in this study was less than 2, 2 to 3.8, and greater than 3.8, indicating favorable, moderate, and high risk of insulin resistance, respectively^24^ In terms of physical activity, the participants were divided into three groups based on the scoring of the International Physical Activity Questionnaire (IPAQ) recommendations for scoring protocol. The groups were low active (< 600 MET–minutes/week), moderate active (≥ 600 MET–minutes/week), and highly active (≥ 3000 MET–minutes/week)^25^ based on the MET-min/wk of walking, moderate-intensity physical activities, and vigorous-intensity physical activities. In terms of fruit and vegetable consumption, the subjects were divided into two groups according to the WHO recommendation, including high consumption (more than 400 g of fruits and vegetables per day) and low consumption (less than 400 g of fruits and vegetables per day)^26^ BMIs between 20 and 24.9 are considered normal; BMIs between 25 and 29.9 are considered overweight; BMIs between 30 and 34.9 are considered obese I; BMIs between 35 and 39.9 are considered obese II; and BMIs ≥ 40 are considered morbid obesity^27^.

### Statistical analysis

All the data were entered in SPSS software version 22 and for descriptive analysis of data, mean and standard deviation or frequency and percentage were used. In order to investigate the relationships due to abnormality, the Mann–Whitney U test and Chi-square test (for classification variables) were used. A multiple logistic regression model was used to determine the factors associated with MtS. The significance level (P value) was considered less than 0.05.

### Ethical code

Prior to the project, the participants signed an informed consent form, and the project protocol was approved by Rafsanjan University of Medical Sciences' local ethical committee (IR.RUMS.REC.1399.050).

All methods were carried out in accordance with relevant guidelines and regulations.

## Results

**Figure 1:**
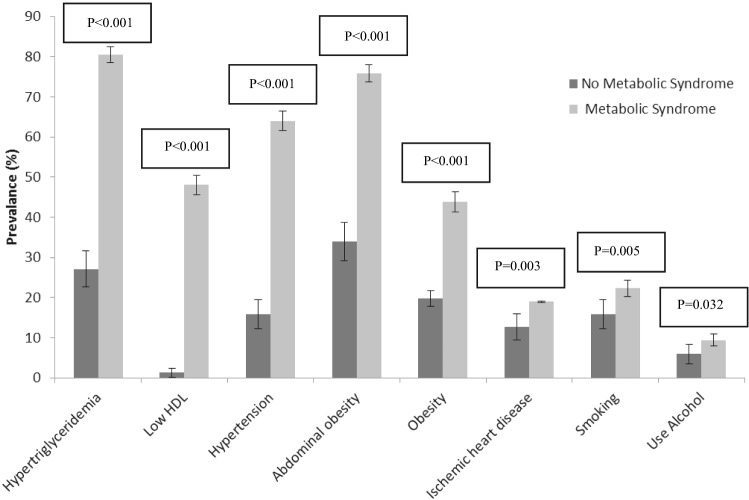
Prevalence (95% confidence interval) of risk factors in the two groups with and without MtS in DM subjects.

Out of 1933 patients with DM in this study, 1213 (62.8%) were female and 720 (37.2%) were male. The participants' average age was 55.92 ± 8.17 years. The prevalence of MtS in this study was estimated to be 80%, with a 95% confidence interval of 78.1–81.8%, meaning that 1546 patients had at least three of the five diagnostic factors of MtS. The prevalence of MtS was significantly higher in women (66.9%) compared to men (33.1%) (*P*value < 0.001). Moreover, the mean age of the subjects in the MtS group was significantly higher than the group without MtS (56.31 ± 7.98 years compared to 54.37 ± 8.74 years, *P* value < 0.001). The frequency distribution comparison of demographic and clinical characteristics of the patients in the two groups with and without MtS is given in Table 1 and Fig. 1.

**Table 1:** A comparison of demographic and clinical characteristics in type 2 diabetes mellitus patients with and without MtS.

Variables		Total (*n* = 1933)	Without metabolic syndrome (*n* = 387)	With metabolic syndrome (*n* = 1546)	*P* value
Age		57 (50–62)	55/5 (47–61)	57 (51–63)	< 0/001
Gender(%)	Male	720 (37/2%)	209 (54%)	511 (33/1%)	< 0/001
	Female	1213 (62/8%)	178 (46%)	1035 (66/9%)	
Marital status(%)	Single	217 (11/2%)	23 (5/9%)	194 (12/5%)	< 0/001
	Married	1716 (88/8%)	364 (94/%1)	1352 (87/5%)	
Education level (%)	Illiterate	353 (18/3%)	48 (12/4%)	305 (19/7%)	< 0/001
	Diploma and lower	1373 (71/0%)	262 (67/7%)	1111 (71/9%)	
	Academic degree	207 (10/7%)	77 (19/9%)	130 (8/4%)	
Family history of ischemic heart disease (%)	Yes	276 (14/3%)	63 (16/3%)	213 (13/8%)	0/208
	No	1657 (85/7%)	324 (83/7%)	1333 (86/2%)	
Family history of heart attack (%)	Yes	223 (11/5%)	45 (11/6%)	178 (11/5%)	0/950
	No	1710 (88/5%)	342 (88/4%)	1368 (88/5%)	
Family history of high blood pressure (%)	Yes	380 (19/7%)	82 (21/2%)	298 (19/3%)	0/397
	No	1553 (80/3%)	305 (78/8%)	1248 (80/7%)	
Family history of diabetes mellitus (%)	Yes	426 (22/0%)	95 (24/5%)	331 (21/4%)	0/183
	No	1507 (78/0%)	292 (75/5%)	1215 (78/6%)	
Family history of stroke (%)	Yes	161 (8/3%)	43 (11/1%)	118 (7/6%)	0/027
	No	1772 (91/%7)	344 (88/9%)	1428 (92/4%)	
Consumption of fruits and vegetables (%)	Low	888 (84/7%)	194 (90/2%)	694 (83/2%)	0/011
	Normal	161 (15/3%)	21 (9/8%)	140 (16/8%)	

Medium (first quarter-third quarter) for age and frequency (%) is reported for qualitative variables. The Mann–Whitney test was used to compare age in the two groups, and the Chi-square test was used for other comparisons.

As can be seen, education level, marital status, smoking, alcohol consumption, history of hypertension, history of ischemic heart disease, family history of stroke, and consumption of fruits and vegetables were significantly different between the two groups (*P* value < 0.05). All the participants were physically low active and most of the subjects (84.7%) were in the group of low consumption of fruits and vegetables.

The prevalence of risk factors in the group without MtS included 33.9% abdominal obesity (95% confidence interval: 29.18–38.62), 19.7% obesity (95% confidence interval: 17.72–21.68), 27.1% hypertriglyceridemia (95% confidence interval: 22.67–31.53), 15.8% hypertension (95% confidence interval: 12.17–19.43), 15.8% smoking (95% confidence interval: 12.17– 19.43), 12.7% history of ischemic heart disease (95% confidence interval: 9.39–16.01), 5.9% alcohol consumption (95% confidence interval: 3.55–8.25), and low HDL 1.3% (95% confidence interval: 0.18–2.42), respectively. The prevalence of risk factors in the MtS group was reported as 80.5% hypertriglyceridemia (95% confidence interval: 78.54–82.46), 75.9% abdominal obesity (95% confidence interval: 73.76–78.04), 64% hypertension (95% confidence interval: 61.66–66.39), 48%low HDL (95% confidence interval: 45.51–50.49), 43.8% obesity (95% confidence interval: 41.33–46.27), 22.3% smoking (95% confidence interval: 20.22–24.38), 19% history of ischemic heart disease (95% confidence interval: 18.80–19.20), and 9.4% alcohol consumption (95% confidence interval: 7.95–10.85), respectively. Figure 1 shows that the prevalence of the aforementioned factors was statistically significant in both groups (*P* value < 0.05).

Given that the studied subjects were all patients with DM, all of them had at least one of the 5 factors of MtS. Therefore, the frequency distribution of the number of MtS factors included 4.4% only one factor (*n* = 85), 15.6% two factors (*n* = 302), 37.1% three factors (i = 716), 31.1% four factors (*n* = 601), and 11.7% five factors (*n* = 226).

Given the abnormal distribution of anthropometric indices and biochemical and laboratory indices, the Mann–Whitney non-parametric test was used for comparing the two groups, which is shown in Table 2.

**Table 2:** A comparison of the median anthropometric and laboratory indices of type 2 diabetes mellitus patients with and without MtS.

Variables	Total (*n* = 1933)	Without metabolic syndrome (*n* = 387)	With metabolic syndrome (*n* = 1546)	*P* value
Waist circumference	99/5 (92/6–106/6)	94/1 (87/1–99/5)	101/2 (94/5–107/9)	< 0/001
BMI	28/66 (25/84–31/93)	26/45 (24/12–29/01)	29/30 (26/40–32/49)	< 0/001
Systolic blood pressure	115 (100–125)	108 (100–118/5)	115 (100–125)	< 0/001
Diastolic blood pressure	70 (65–80)	70(65–80)	75 (65–80)	< 0/001
Heart rate	76 (70–82)	74 (68–80)	76 (70–83)	0/001
Fasting blood sugar	144 (114–188)	143 (111–182/25)	145 (115–189)	0/3
Triglyceride	165 (121–229)	126 (100–160)	179 (132/5–240)	< 0/001
Cholesterol	193 (167–223)	191/5 (164–218)	194 (167–225)	0/098
HDL	56 (49–64)	59 (52–66)	56 (48–63)	< 0/001
LDL	100 (78–124)	99 (80–125)	100 (78–124)	0/430
Creatinine	1 (0/9–1/2)	1 (0/9–1/2)	1 (0/9–1/2)	0/143
BUN	14 (12–17)	14 (12–17)	14 (12–17)	0/261
ALP	232 (192–277)	219 (186/75–259)	235 (194–281)	< 0/001
TG to HDL ratio	2/96 (2/07–4/29)	2/15 (1/60–2/84)	3/22 (2/25–4/49)	< 0/001

A median (first quarter-third quarter) is reported for the variables. A Mann–Whitney test was used to compare the two groups. *LDL* Low-density lipoproteins, *HDL* High-density lipoproteins, *ALP* Alkaline phosphatase, and *BUN* Blood urea nitrogen.

The median of BMI, systolic blood pressure, diastolic blood pressure, heart rate, triglyceride, ALP, and TG to HDL ratio were significantly higher in the MtS group, and HDL was significantly lower than the group without MtS.

In order to investigate the relationship between demographic characteristics, disease history, anthropometric indices, and biochemical factors with MtS, univariate logistic regression was performed for all the studied variables. Then, significant variables at the level of 0.1 were entered into the multiple logistic regression model using the Backward LR method. In this study, smoking, alcohol consumption, TG to HDL ratio, abdominal obesity, and hypertension were identified as factors associated with MtS based on the results of the logistic regression model (Table 3).

**Table 3:** Multivariate logistic regression results.

Variables		OR (95% CI)	*P* value
TG to HDL ratio		1/42 (1/30–1/55)	< 0/001
Abdominal obesity	No	ref	–
	Yes	13/73 (9/77–19/29)	< 0/001
Smoking	No	ref	–
	Yes	5/60 (3/67–8/55)	< 0/001
Alcohol consumption	No	ref	–
	Yes	4/098 (2/32–7/23)	< 0/001
Hypertension	No	ref	–
	Yes	13/54 (9/55–19/19)	0/011

Multiple logistic regression model using the Backward LR method. *HDL* High-density lipoproteins, *TG* Triglycerides.

As a result, smoking increased the risk of MtS by 5.60% (95% confidence interval: 3.67–8.55), alcohol consumption by 4.1% (95% confidence interval: 2.32–7.23), the TG to HDL ratio by 1.42% (95% confidence interval: 1.30–1.55), abdominal obesity by 13.73% (95% confidence interval: 9.77–19.29), and hypertension by 13.54% (95% confidence interval: 9.55–19.19). Given that hypertension and abdominal obesity are two of the five causes of MtS, it is associated with an increased risk of developing MtS.

## Discussion

Cardiovascular diseases are the leading cause of death and disability in diabetic patients^28^ Metabolic syndrome is defined by the coexistence of several classic cardiovascular risk factors, such as insulin resistance, hypertension, high triglycerides, and high-density lipoprotein (HDL) cholesterol^29^ In this syndrome, some biomarkers differ, which can predict a future event. Chronic mild inflammation biomarkers (e.g. Creative protein, CRP), increased oxidant stress (e.g. oxidized low density lipoprotein, LDL), thrombophilia (e.g. plasminogen activator inhibitor 1, PAI1), and endothelial dysfunction (e.g. Eselectin) may all increase the risk of atherosclerosis and clinical cardiovascular events^30,31^ Therefore, MtS is a set of multiple risk factors for atherosclerotic cardiovascular disease^32^ MtS is strongly associated with DM. In this type of diabetes, there is insulin resistance with secondary hyperinsulinemia, and it is often associated with high blood pressure, dyslipidemia, atherosclerosis, and most importantly, obesity, especially central obesity. The etiology of MtS consists of separate components of the MtS (such as hypertension, DM, dyslipidemia) causing complex conditions^33^ The prevalence of MtS in this study was estimated to be 80%. Its prevalence according to IDF criteria in people with Type DM in the study by Moreira et al. was reported to be 74.3% *. It was also 69.5% in the study by AlSaraj et al.^34^ Different prevalence of MtS in populations may be due to nutritional, epidemiological, and demographic transitions^35^, as well as ethnic^36^ social, and environmental^37^ disparities in addition to methodological differences..

In this study, the prevalence of hypertension in the group without MtS was 15.8%, and in the MtS group it was 64%. Previous studies have shown the relationship between metabolic syndrome and high blood pressure^33^ The prevalence of metabolic syndrome is higher in hypertensive patients than in the general population, and there is an increasing prevalence of MtS in newly diagnosed hypertensive patients, with increased blood pressure being a marker for MtS^38^ This study also showed that in DM patients, the prevalence of hypertension is higher in MtS and recognizing this fact has led to a change in managing high blood pressure and other CVD risk factors.

In the current study, smoking, alcohol consumption, and TG to HDL ratio were also identified as factors associated with MtS in patients with DM. These results were confirmed by Lindsay et al. and Mottillo et al.^39,40^ According to Slagter et al., alcohol and cigarette use increase the risk of MtS and some of its components in a dose-dependent manner^41^ It has been found that high levels of LDL, high blood pressure, and smoking are the most important risk factors for cardiovascular disease in DM. Low HDL cholesterol, insulin resistance, hyperglycemia, and inflammation are also predictors of cardiovascular complications^42^ Sadabadi F et al. reported that elevated fasting glucose levels are strongly associated with cardiovascular events in this population^43^ The differences in this study could also be attributed to the previously mentioned socio-demographic or lifestyle factors.

Previous research has found that obesity, particularly central obesity, is the primary underlying cause of MtS, resulting in a genetic predisposition to other risk factors like dyslipidemia and hypertension^44^. In the present study, the prevalence of abdominal obesity in diabetic patients without MtS was 33.9%, which reached 75.9% in DM patients with MtS. In the study by Cheng et al., abdominal obesity was reported in 91.6% of people with DM^45^ which is higher than the results of the present study. Central obesity is a major risk factor for MtS and DM^46^ and also increases the risk of dyslipidemia and coronary artery disease^47^.

The most important limitations of this study were the lack of access to complete information in the files and the defects in the files, which were controlled as much as possible by removing incomplete files and replacing other patients, to the extent that the sample size and analysis of the study were not affected. The investigation of the Rafsanjan cohort population was one of our study's strong points, as the results can be generalized to the entire Rafsanjan population.

## Conclusion

The high prevalence of MtS in patients with DM in the Rafsanjan adult cohort study indicates the importance of this issue. Therefore, high-risk patients can be identified using MtS screening in primary health centers, and they can benefit from timely multifactorial interventions. Some suggested solutions include reducing smoking and alcohol consumption, as well as controlling hypertension, hyperlipidemia, and obesity.

## Data Availability

Correspondence and requests for materials should be addressed to Gh B. or Z.K.

## References

[1] Garhwal S, Poonia AK, Agarwal V (2020). Study of effect of diabetes mellitus on classical risk factors for coronary artery disease. Int. J. Res. Med.Sci..

[2] Esteghamati A, Larijani B, Aghajani MH, Ghaemi F, Kermanchi J, Shahrami A (2017). Diabetes in Iran: Prospective analysis from first nationwide diabetes report of National Program for Prevention and Control of Diabetes (NPPCD-2016). Sci. Rep..

[3] Sarwar N, Gao P, Kondapally Seshasai S, Gobin R, Kaptoge S, Di Angelantonio E (2010). Diabetes mellitus, fasting blood glucose concentration, and risk of vascular disease: A collaborative meta-analysis of 102 prospective studies. Lancet.

[4] Haffner SM, Lehto S, Rönnemaa T, Pyörälä K, Laakso M (1998). Mortality from coronary heart disease in subjects with type 2 diabetes and in nondiabetic subjects with and without prior myocardial infarction. NEJM.

[5] Martín-Timón I, Sevillano-Collantes C, Segura-Galindo A, del Cañizo-Gómez FJ (2014). Type 2 diabetes and cardiovascular disease: Have all risk factors the same strength?. World J. Diabetes..

[6] Straka RJ, Liu LZ, Girase PS, DeLorenzo A, Chapman RH (2009). Incremental cardiovascular costs and resource use associated with diabetes: An assessment of 29,863 patients in the US managed-care setting. Cardiovasc. Diabetol..

[7] Patel N (2017). Impact of diabetes on heart failure incidence in adults with ischemic heart disease. JDC..

[8] Mohammadi M, Mirzaei M, Karami M (2018). Potential impact fraction of ischemic heart disease associated with diabetes mellitus in Yazd-Iran. Irje..

[9] Mohamadshahi M, Veissi M, Haidari F, Shahbazian H, Kaydani G-A, Mohammadi F (2014). Effects of probiotic yogurt consumption on inflammatory biomarkers in patients with type 2 diabetes. BI..

[10] Echouffo-Tcheugui JB, Kengne AP (2013). On the importance of global cardiovascular risk assessment in people with type 2 diabetes. Prim. Care Diabetes..

[11] Gæde P, Vedel P, Larsen N, Jensen GV, Parving H-H, Pedersen O (2003). Multifactorial intervention and cardiovascular disease in patients with type 2 diabetes. NEJM.

[12] Del Cañizo Gómez FJ, Andrés MNM (2008). Strict control of modifiable cardiovascular risk factors in patients with type 2 diabetes mellitus. Med. Clin..

[13] Vergès B (2020). Cardiovascular disease in type 1 diabetes: A review of epidemiological data and underlying mechanisms. Diabetes Metab..

[14] Wan S-H, Chen HH, Wan S-H, Chen HH (2022). Precision medicine for diabetes and cardiovascular disease. Precision medicine Diabetes.

[15] Bovolini A (2021). Metabolic syndrome pathophysiology and predisposing factors. Int J. Sports Med..

[16] Alberti K (2009). Harmonizing the metabolic syndrome: A joint interim statement of the international diabetes federation task force on epidemiology and prevention; national heart, lung, and blood institute; American heart association; world heart federation; international atherosclerosis society; and international association for the study of obesity. Circulation.

[17] Yadav D, Mahajan S, Subramanian SK, Bisen PS, Chung CH, Prasad G (2013). Prevalence of metabolic syndrome in type 2 diabetes mellitus using NCEP-ATPIII, IDF and WHO definition and its agreement in Gwalior Chambal region of Central India. Glob. J. Health Sci..

[18] Pokharel DR (2014). Prevalence of metabolic syndrome in Nepalese type 2 diabetic patients according to WHO, NCEP ATP III IDF Harmonized criteria. JDMDC..

[19] Eckel RH, Bornfeldt KE, Goldberg IJ (2021). Cardiovascular disease in diabetes, beyond glucose. Cell. Metab..

[20] Ahmed I, Goldstein BJ (2006). Cardiovascular risk in the spectrum of type 2 diabetes mellitus. Mt. Sinai. J. Med..

[21] Hakimi H (2021). The profile of Rafsanjan cohort study. Eur. J. Epidemiol..

[22] Poustchi H (2018). Prospective epidemiological research studies in Iran (the Persian cohort study): Rationale, objectives, and design. Am. J. Epidemiol..

[23] Grundy SM (2006). Diagnosis and management of the metabolic syndrome: An American heart association/national heart, Lung, and blood institute scientific statement. Curr. Opin. Cardiol..

[24] Scicali R (2021). High TG to HDL ratio plays a significant role on atherosclerosis extension in prediabetes and newly diagnosed type 2 diabetes subjects. Diabetes Metab. Res. Rev..

[25] Cheng H. (2016) A simple, easy-to-use spreadsheet for automatic scoring of the international physical activity questionnaire (IPAQ) Short form (updated November 2016). ResearchGate, editor.

[26] Who J, Consultation FE (2003). Diet, nutrition and the prevention of chronic diseases. World Health Organ. Tech. Rep. Ser..

[27] Kabiru W, Raynor BD (2004). Obstetric outcomes associated with increase in BMI category during pregnancy. AJOG..

[28] Matheus ASdM, Tannus LRM, Cobas RA, Palma CCS, Negrato CA, (2013) Gomes MdB. Impact of diabetes on cardiovascular disease: An update. Int. J. Hypertens 653789 10.1155/2013/653789 PMC3603160 23533715

[29] Fargion S, Porzio M, Fracanzani AL (2014). Nonalcoholic fatty liver disease and vascular disease: State-of-the-art. WJG..

[30] Bonora E (2006). "The metabolic syndrome and cardiovascular disease. Ann. Med..

[31] Hajar R (2017). Risk factors for coronary artery disease: Historical perspectives. Heart Views..

[32] Grundy SM (2016). Metabolic syndrome update. Trends Cardiovasc. Med..

[33] Duvnjak L, Bulum T, Metelko Z (2008). Hypertension and the metabolic syndrome. Diabetol. Croat..

[34] AlSaraj F (2009). Prevalence of the metabolic syndrome in patients with diabetes mellitus. Ir. J. Med. Sci..

[35] Amuna P, Zotor FB (2008). Epidemiological and nutrition transition in developing countries: Impact on human health and development: The epidemiological and nutrition transition in developing countries: Evolving trends and their impact in public health and human development. Proc. Nutr. Soc..

[36] Salsberry PJ, Corwin E, Reagan PB (2007). A complex web of risks for metabolic syndrome: Race/ethnicity, economics, and gender. Am. J. Prev. med..

[37] Chow CK, Lock K, Teo K, Subramanian S, McKee M, Yusuf S (2009). Environmental and societal influences acting on cardiovascular risk factors and disease at a population level: A review. Int. J. Epidemiol..

[38] Govindula A, Vlupadas C, Panchagiri S (2016). Prevalence of metabolic syndrome in hypertensive de novo patients at a tertiary care hospital. Indian J. Pharm. Pract..

[39] Lindsay RS, Howard BV (2004). Cardiovascular risk associated with the metabolic syndrome. Curr. Diab. Rep..

[40] Mottillo S (2010). The metabolic syndrome and cardiovascular risk: A systematic review and meta-analysis. J. Am. Coll. Cardiol..

[41] Slagter SN (2014). Combined effects of smoking and alcohol on metabolic syndrome: The LifeLines cohort study. PLoS ONE.

[42] Laakso M, Kuusisto J (2014). Insulin resistance and hyperglycaemia in cardiovascular disease development. Nat. Rev. Endocrinol..

[43] Sadabadi F, Gholoobi A, Heidari-Bakavol A, Mouhebati M, Javandoost A, Asadi Z, Saberi-Karimian M, Darroudi S, Mohebbseraj MS, Rahmani F, Gonabadi NM (2020). Decreased threshold of fasting serum glucose for cardiovascular events: MASHAD cohort study. Rep. Biochem. Mol. Biol..

[44] Fahed G (2022). Metabolic syndrome: Updates on pathophysiology and management in 2021. Int. J. Mol. Sci..

[45] Cheng Y (2014). Cardiometabolic risk profiles associated with chronic complications in overweight and obese type 2 diabetes patients in South China. PLoS ONE.

[46] Tyrovolas S (2015). Diabetes mellitus and its association with central obesity and disability among older adults: A global perspective. Exp. Gerontol..

[47] Onat A, Avcı GŞ, Barlan M, Uyarel H, Uzunlar B, Sansoy V (2004). Measures of abdominal obesity assessed for visceral adiposity and relation to coronary risk. Int. J. Obes..

